# A systematic review of symptomatic small bowel lipomas of the jejunum and ileum

**DOI:** 10.1016/j.amsu.2020.08.028

**Published:** 2020-09-01

**Authors:** Nicholas Farkas, Joshua Wong, Jordan Bethel, Sherif Monib, Adam Frampton, Simon Thomson

**Affiliations:** West Hertfordshire Hospitals NHS Trust, Vicarage Rd, Watford, Hertfordshire, WD18 0HB, UK

**Keywords:** Lipoma, Small bowel, Small intestine, Jejunum, Ileum

## Abstract

**Introduction:**

Small bowel lipomas are rarely encountered benign adipose growths found within the small intestine wall or mesentery. Limited up-to-date evidence exists regarding such lipomas. We aim to aid clinical decision-making and improve patient outcomes through this comprehensive review.

**Methodology:**

The terms ‘small bowel,’ ‘small intestine,’ ‘jejunum’ and ‘ileum’ were combined with ‘lipoma.’ EMBASE, Medline and PubMed database searches were performed. All papers published in English from 01/01/2000-31/12/2019 were included. Simple statistical analysis (*t*-test, Anova) was performed.

**Results:**

142 papers yielded 147 cases (adults = 138, pediatric = 9). Male = 88, female = 59 (average age = 49.9 years). Presenting symptoms: abdominal pain = 68.7%; nausea/vomiting = 35.3%, hematochezia/GI bleeding = 33.3%; anaemia = 10.9%; abdominal distension = 12.2%; constipation = 8.9%; weight loss = 7.5%. Mean preceding symptom length = 58.1 days (symptoms >1 year excluded (n = 9)). Diagnostic imaging utilised: abdominal X-Ray = 33.3%; endoscopy = 46.3%; CT = 78.2%; ultrasound = 23.8%. 124/137 (90.5%) required definitive surgical management (laparotomy = 89, laparoscopcic = 35). 9 patients were successfully managed endoscopically. Lipoma location: ileum = 59.9%, jejunum = 32%, mesentery = 4.8%. Maximal recorded lipoma size ranged 1.2–22 cm.

Mean maximum lipoma diameter and management strategy comparison: laparotomy 5.6 cm, laparoscopic = 4.4 cm, endoscopic = 3.7 cm, conservative = 4.5 cm. One-way Anova test, p value = 0.21. Average length of stay (LOS) was 7.4 days (range = 2–30). T-test p value = 0.13 when comparing management modalities and LOS. 4 complications, 0 mortality.

**Conclusions:**

Important previously undocumented points are illustrated; a clearer symptom profile, diagnostic investigations utilised, size and site of lipomas, types and effectiveness of management modalities, associated morbidity and mortality. Open surgery remains the primary management. No statistically significant difference in LOS and lipoma size is demonstrated between management strategies. Endoscopic and laparoscopic techniques may reduce utilising invasive surgery in the future as skillset and availability improve.

## Highlights

• Up to date overview of symptomatic lipomas of the jejunum and ileum which no other paper has previously covered.
• Highlights associated symptom profile, investigations, site and size of symptomatic lipomas, morbidity and mortality.
• Evaluates the effectiveness of management strategies.
• Offers a practical summary that may help guide other clinicians faced with similar presentations in the future.

## 1 Introduction

Little up to date evidence exists regarding lipomas of the small bowel other than anecdotal case reports. Much of the data quoted by these papers can be traced back to epidemiological studies carried out over 20 years ago. More recent studies relate to reviews of duodenal [1] and colonic [2] lipomas. However, no current systematic review exists pertaining to symptomatic lipomas of the ileum and jejunum, which for the purposes of this paper we shall refer to as small bowel lipomas.

Small bowel lipomas are rarely encountered benign adipose growths found within the wall or mesentery of the small intestine. Incidence of intestinal lipomas ranges from 0.035% to 4.4% [3]. Lipomas can arise throughout the gastrointestinal tract with the small bowel accounting for 25% [4]. These benign tumors arise from the sub mucosa of the small intestine in 90% of cases [5]. Small bowel lipomas are most commonly found incidentally with the majority of patients being asymptomatic.

Unlike more proximal and distal lesions that can be easily accessed and investigated with endoscopy, small bowel tumors represent a difficult diagnostic entity. The clinical picture is often not clear, with vague symptoms commonly reported.

This paper comprehensively reviews symptomatic small bowel lipomas, including demographics, clinical presentation, diagnostic investigations, management, pathology, length of stay and mortality. We hope clinicians managing such patients can draw on this paper to aid clinical decision-making and improve patient outcomes.

## 2 Methodology

The search terms ‘small bowel,’ ‘small intestine,’ ‘jejunum’ and ‘ileum’ were combined with ‘lipoma.’ Multiple database searches of EMBASE, Medline and PubMed were conducted. All papers published from January 01, 2000 to December 31, 2019 in English were included. Hand searches were also performed using Google Scholar with the same search terms. The first 50 hand search results were included for screening. The paper was registered with the International Prospective Register of Systematic Reviews, (PROSPERO) CRD42020172916.

Simple statistical analysis was preformed where appropriate. T-test was undertaken to compare length of stay between open and laparoscopic surgery patients. One-way Anova test was used for comparison of lipoma size between patients undergoing different management modalities (open surgery, laparoscopic surgery, endoscopic management).

**Fig. 1:**
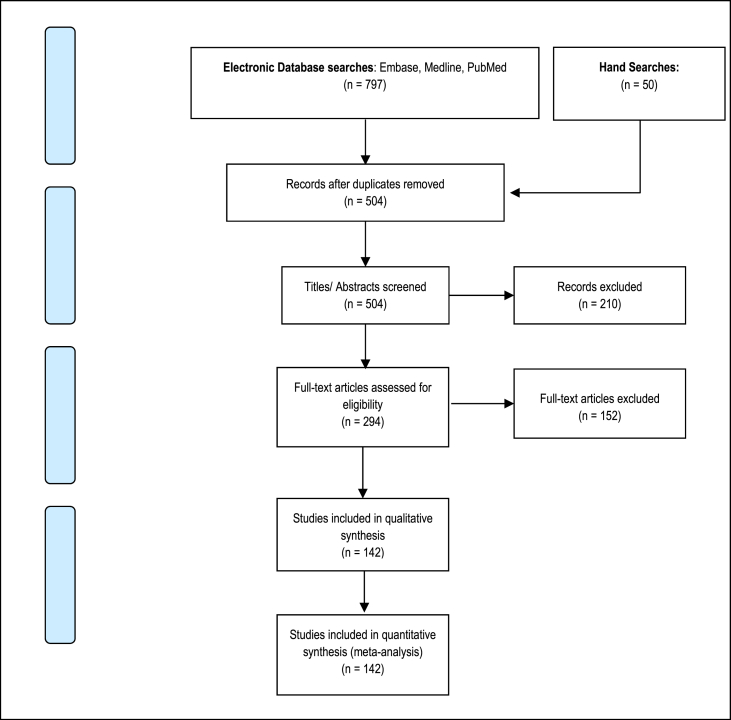
Prisma diagram of database searches.

Two reviewers independently analysed the searches, abstracts and papers identified to reduce bias. The PRISMA [6] diagram (Fig. 1) demonstrates our search strategy. The selected papers were analysed for multiple outcomes relating to sex, age, presenting complaint, diagnostic modalities, management strategies, complications, mortality and length of stay. The AMSTAR 2 and PRISMA guidelines for assessing methodological quality in systematic reviews were followed [7].

Of the 797 papers derived from database and hand searches, 504 titles/abstracts remained once duplicates had been removed. These were screened with a further 210 papers excluded because they were not relevant to the paper. Two independent reviewers then reviewed 294 full-text articles. A further 152 papers were excluded; 103/152 were either abstract only (full text not accessible or published), in a different language or not case specific; 18/152 related to duodenal lipomas; 5/152 were incidental lipomas in asymptomatic patients; the remaining 26/152 were unable to be accessed. Thus a total of 142 were included yielding a total of 147 cases.

All papers related to individual cases or case series. Given the observational nature of such reports and that no randomised control trials were included, reporter and publication bias was deemed to be low. No funding or other financial support was received in relation to this study.

## 3 Results

138 adults and 9 pediatric (age 0–16) cases were recorded. Average age was 49.9 (2–87) years. Male number (n) = 88, female n = 59, male:female ratio was 1.49:1. Average age was male = 49yrs, female = 51.4yrs (Table 1).

**Table 1:** Results.

Author	Year	Title	Journal	Age	Sex	Symptoms	Length of symptoms	Site of lipoma	Largest diameter (cm)	Emergency (Y/N)	Definitive management	Length of stay (days)
**Abbasakoor et al.**	2010	Midgut pain due to an intussuscepting terminal ileal lipoma: A case report	Journal of Medical Case Reports	52	F	Abdominal pain, constipation	3 months	Ileum	4	N	Laparoscopic	4
**Abdelmohsen et al.**	2019	An ileo-ileal intussusception secondary to polypoid lipoma in a child, a case report and review of the literature	International Journal of Surgery Case Reports	4	M	Abdominal pain, vomiting	24 h	Ileum	4	Y	Laparotomy	7
**Ahmed et al.**	2004	Acute abdomen from a Meckel lipoma	Journal of the Royal Society of Medicine	28	M	Abdominal pain, GI bleed, vomiting, diarrhoea	24 h	Jejunum	3	Y	Laparotomy	Not stated
**Ahmed et al.**	2018	Submucosal Lipomas Causing Intussusception and Small Bowel Obstruction: A Case Report	Cureus Journal of Medical Science	67	M	Abdominal pain, nausea, vomiting, constipation	5 days	Ileum	Not stated	Y	Laparoscopic	Not stated
**Akagi et al.**	2008	Adult intussusception caused by an intestinal lipoma: Report of a case	Journal of Nippon Medical School	36	M	Abdominal pain, nausea, vomiting, abdominal distension	24 h	Ileum	4	Y	Laparotomy	15
**Akimaru et al.**	2006	Resection of over 290 polyps during emergency surgery for four intussusceptions with Peutz-Jeghers syndrome: Report of a case	Surgery Today	41	M	Abdominal pain, nausea	12 h	Ileum	2	Y	Laparotomy	Not stated
**Ako et al.**	2010	Laparoscopic resection of ileal lipoma diagnosed by multidetector-row computed tomography	Surgical Laparoscopy, Endoscopy and Percutaneous Techniques	43	F	Abdominal pain, nausea	6 h	Ileum	2.4	Y	Laparoscopic	9
**Al-Radaideh et al.**	2018	Adult intussusception: A 14-year retrospective study of clinical assessment and computed tomography diagnosis	Belgian Acta Gastro-Enterologica Belgica	43	F	Abdominal pain	Not stated	Jejunum	13	Y	Not stated	Not stated
**Alsayegh et al.**	2019	Mesenteric lipoma presenting as small bowel volvulus	Journal of Pediatric Surgery Case Reports	4	F	Abdominal pain, vomiting	Not stated	Mesentery	7.6	Y	Laparotomy	Not stated
**Asaumi et al.**	2014	Pediatric ileoileal intussusception with a lipoma lead point: a case report	Gastroenterology Report (Oxford Academic)	7	M	Abdominal pain	3 days	Ileum	Not stated	Y	Laparotomy	8
**Atila et al.**	2007	Symptomatic intestinal lipomas requiring surgical interventions secondary to ileal intussusception and colonic obstruction: Report of two cases	Turkish National Journal of Trauma and Emergency Surger	47	F	Abdominal pain, nausea	2 days	Ileum	5	Y	Laparotomy	Not stated
**Bakker et al.**	2009	Nausea caused by a jejunal lipoma	Clinical Gastroenterology and Hepatology	57	F	Nausea, vomiting, weight loss	2 years	Jejunum	10	N	Laparotomy	Not stated
**Balmadrid et al.**	2014	Chronic iron deficiency anemia caused by small-bowel lipoma	Gastrointestinal Endoscopy	64	M	Fatigue, anaemia	1 year	Ileum	1.9	N	Laparoscopic	Not stated
**Bilgin et al.**	2012	Ileocecal Intussusception due to a Lipoma in an Adult	Case Reports in Surgery	39	F	Abdominal pain	24 h	Ileum	2.5	N	Laparotomy	7
**Bosman et al.**	2014	Ileocaecal intussusception due to submucosal lipoma in a pregnant woman	British Medical Journal Case Reports	30	F	Abdominal pain, nausea, vomiting	2 days	Ileum	1.5	Y	Laparotomy	5
**Chehade et al.**	2015	Large ileocecal submucosal lipoma presenting as hematochezia, a case report and review of literature	International Journal of Surgery Case Reports	42	F	Abdominal pain, GI bleeding	2 months	Ileocaecal valve	4.5	Y	Laparotomy	Not stated
**Cherian et al.**	2004	Small bowel volvulus due to giant mesenteric lipoma	Pediatric Surgery International	14	F	Abdominal pain, vomiting	8 h	Mesentery	16	Y	Laparotomy	Not stated
**Chou et al.**	2008	Obscure gastrointestinal bleeding caused by small bowel lipoma	Internal Medicine	57	M	GI bleed	5 days	Ileum	3	Y	Laparotomy	Not stated
**Cuciureanu et al.**	2019	Ulcerated intussuscepted jejunal lipoma-uncommon cause of obscure gastrointestinal bleeding: A case report	World Journal of Clinical Cases	63	M	Abdominal pain, nausea, anaemia	Not stated	Jejunum	6	Y	Laparotomy	14
**Devillers et al.**	2016	An atypical acute small-bowel obstruction	Diagnostic and Interventional Imaging	54	F	Abdominal pain, vomiting	24 h	Mesentery	Not stated	Y	Laparotomy	Not stated
**Di Saverio et al.**	2010	Concomitant intestinal obstruction: a misleading diagnostic pitfall	British Medical Journal Case Reports	78	F	Constipation	2 months	Ileum	3	Y	Laparotomy	9
**Duijff et al.**	2007	Intussusception in adults: report of four cases and review of the literature	Case Reports in Gastroenterology	42	M	Abdominal pain	Several months	Ileocaecal valve	3	Y	Laparotomy	7
**Dultz et al.**	2009	Ileocecal valve lipoma with refractory hemorrhage	Journal of the Society of Laparoendoscopic Surgeons	77	M	GI bleed	2 days	Ileocaecal valve	3.5	Y	Laparoscopic	5
**Ertem et al.**	2010	Application of laparoscopy in the management of obscure gastrointestinal bleeding	Surgical Laparoscopy, Endoscopy and Percutaneous Techniques	47	M	GI bleed	Not stated	Jejunum	Not stated	Y	Laparoscopic	7
**Eyselbergs et al.**	2014	Ileocolic intussusception due to lipomatosis of the ileum: A common complication of a rare clinical entity	Journal of the Belgian Society of Radiology	56	M	Abdominal pain, GI bleeding	Not stated	Ileum	2	Y	Laparotomy	Not stated
**Feo et al.**	2019	A rare case of ileo-ileal intussusception due to a bleeding lipomatous mass treated by laparoscopic ileal resection	Italian annals of Surgery	69	M	GI bleed	1 h	Ileum	3	Y	Laparoscopic	3
**Ferrara et al.**	2012	Laparoscopic resection of small bowel lipoma causing obscure gastrointestinal bleeding	Updates in Surgery	78	F	GI bleed	1 h	Jejunum	3	Y	Laparoscopic	Not stated
**Gao et al.**	2014	Ileo-colonic intussusception secondary to small-bowel lipomatosis: A case report	World Journal of Gastroenterology	52	F	Abdominal pain	21 days	Ileum	5	Y	Laparotomy	30
**Garcia Zamora et al.**	2014	Intestinal intussusception due to a lipoma in Meckel's diverticulum	Spanish Surgery	50	M	Abdominal pain, vomiting	1 year	Meckel's diverticulum	5	N	Laparotomy	Not stated
**Hanafiah et al.**	2019	Adult entero-enteric intussusception secondary to lipoma	Clinical Case Reports	35	M	Abdominal pain, vomiting	Not stated	Ileum	2	Y	Laparotomy	Not stated
**Hasab Allah et al.**	2013	Percutaneous ultrasound-guided bowel wall core biopsy: A nonconventional way of diagnosis of gastrointestinal lesions	Surgical Endoscopy	61	F	Not stated	Not stated	Jejunum	2	Y	Not stated	Not stated
**Honda et al.**	2012	Enteroscopic and radiologic diagnoses, treatment, and prognoses of small-bowel tumors	Gastrointestinal Endoscopy	61	F	GI bleed	Not stated	Jejunum	Not stated	Y	Not stated	Not stated
				61	M	GI bleed	Not stated	Jejunum	Not stated	Y	Endoscopic	Not stated
				61	M	GI bleed	Not stated	Jejunum	Not stated	Y	Endoscopic	Not stated
**Hou et al.**	2012	Laparoscopic management of small-bowel intussusception in a 64-year-old female with ileal lipomas	World Journal of Gastrointestinal Surgery	64	F	Abdominal pain	2 h	Ileum	Not stated	Y	Laparoscopic	7
**Javia et al.**	2016	Endoscopic resection of small-bowel submucosal nodule	Endoscopy	67	F	Evaluation after positive faecal immunochemical testing	Not stated	Ileum	2	N	Endoscopic	Not stated
**Jayasundara et al.**	2016	A case of gastroduodenal lipomatosis	Annals of the Royal College of Surgeons of England	43	F	Constipation, vomiting	5 days	Jejunum	Not stated	Y	Laparotomy	5
**Jiang et al.**	2015	Submucosal Lipoma: a Rare Cause of Recurrent Intestinal Obstruction and Intestinal Intussusception	Journal of Gastrointestinal Surgery	50	M	Abdominal pain	1 month	Ileum	4	N	Laparotomy	8
**Jung et al.**	2007	Intestinal chondrolipoma: uncommon cause of bowel obstruction	Journal of Pediatric Surgery	11	M	Abdominal pain, abdominal distension, vomiting.	4 h	Jejunum	7.5	Y	Laparotomy	7
**Kaczynski et al.**	2012	Giant lipoma of the small bowel associated with perforated ileal diverticulum	British Medical Journal Case Reports	38	M	Abdominal pain, weight loss, nausea, vomiting, fever	72 h	Ileum	9	Y	Laparotomy	4
**Kakiuchi et al.**	2017	A small intestine volvulus caused by strangulation of a mesenteric lipoma: a case report	Journal of Medical Case Reports	67	M	Abdominal pain, nausea, vomiting	Not stated	Ileum	10	Y	Laparoscopic	6
**Kamaoui et al.**	2007	Jejunojejunal intussusception secondary to a lipoma	French Radiology Sheets	55	M	Abdominal pain, anaemia, GI bleed	3 months	Jejunum	4	Y	Laparotomy	7
**Kane et al.**	2019	Gastrointestinal hemorrhage caused by small intestinal benign tumors: 2 cases report	Pan African Medical Journal	72	M	Fatigue, GI bleed, anaemia	Not stated	Jejunum	Not stated	Y	Not stated	Not stated
	2019	Gastrointestinal hemorrhage caused by small intestinal benign tumors: 2 cases report	Pan African Medical Journal	68	M	GI bleed, anaemia	Not stated	Ileum	Not stated	Y	Endoscopic	Not stated
**Kang et al.**	2014	Resolution of intussusception after spontaneous expulsion of an ileal lipoma per rectum: A case report and literature review	World Journal of Surgical Oncology	65	F	Abdominal pain, nausea	5 days	Not stated	7	Y	Conservative	Not stated
**Karadeniz Cakmak et al.**	2007	Lipoma within inverted Meckel's diverticulum as a cause of recurrent partial intestinal obstruction and hemorrhage: a case report and review of literature	World Journal of Gastroenterology	47	M	Abdominal pain, constipation, fatigue	4 months	Meckel's diverticulum	4	Y	Laparotomy	5
**Karthikeyan et al.**	2012	Jejuno-jejunal intussusception secondary to small-bowel lipomatosis: a case report	South African Journal of Surgery	60	M	Abdominal pain, vomiting, abdominal distension	3 days	Jejunum	Not stated	Y	Laparotomy	10
**Katergiannakis et al.**	2004	Jejunojenulal intussusception due to an intraluminal lipoma	Annals of Gastroenterology	55	M	Abdominal pain, GI bleeding, anaemia	3 months	Jejunum	4	Y	Laparotomy	7
**Kenkare et al.**	2010	Macrodactylia fibrolipomatosis presenting as a small bowel obstruction	Southern Medical Journal	69	M	Abdominal pain, abdominal distension, vomiting	Not stated	Jejunum	3.7	Y	Laparotomy	Not stated
**Kida et al.**	2017	A unique case of massive gastrointestinal bleeding	SAGE Open Medical Case Reports	67	M	GI bleed, anaemia	Not stated	Jejunum	4	Y	Laparotomy	Not stated
**Kim et al.**	2013	A case of jejunal lipomatosis diagnosed with double-balloon enteroscopy	Journal of Gastroenterology and Hepatology Research	50	M	Abdominal pain	3 months	Jejunum	Not stated	Y	Conservative	Not stated
**Kim et al.**	2017	Spontaneous peeled ileal giant lipoma caused by lower gastrointestinal bleeding A case report	Medicine (United States)	82	F	Abdominal pain, GI bleed	7 days	Ileum	3	Y	Laparoscopic	8
**Kiziltas et al.**	2009	A remarkable intestinal lipoma case	Turkish Journal of Trauma and Emergency Surgery	37	F	Abdominal pain, nausea, vomiting, obstruction, anaemia	Not stated	Jejunum	4	Y	Laparotomy	Not stated
**Komagata et al.**	2007	Extensive lipomatosis of the small bowel and mesentery: CT and MRI findings	Journal of Medical Imaging and Radiation Oncology	49	F	Abdominal pain, abdominal distension	Long term	Ileum	2	Y	Conservative	Not stated
**Konik et al.**	2018	Complete small bowel obstruction without intussusception due to a submucosal lipoma	Journal of Surgical Case Reports	53	F	Abdominal pain, abdominal distension, nausea, vomiting	1 day	Jejunum	1.5	Y	Laparotomy	18
**Kraniotis et al.**	2016	Giant ileocolic intussusception in an adult induced by a double ileal lipoma: a case report with pathologic correlation	Radiology Case Reports	30	M	Abdominal pain, nausea and vomiting	3 days	Ileum	3	Y	Laparotomy	Not stated
**Krasniqi et al.**	2011	Compound double ileoileal and ileocecocolic intussusception caused by lipoma of the ileum in an adult patient: A case report	Journal of Medical Case Reports	46	M	Abdominal pain, nausea, vomiting	4 months	Ileum	3.5	Y	Laparotomy	30
**Krespis et al.**	2006	Partial intestinal obstruction caused by a lipoma within a Meckel's diverticulum	Digestive and Liver Disease	47	M	Abdominal pain, fatigue, constipation, GI bleed	4 months	Meckel's diverticulum	5	y	Laparotomy	5
**Kroner et al.**	2015	Endoscopic Mucosal Resection of Jejunal Polyps using Double-Balloon Enteroscopy	GE Portuguese Journal of Gastroenterology	58	F	GI bleed	Not stated	Jejunum	2	N	Endoscopic	Not stated
**Kumar et al.**	2017	Rare diagnosis of intestinal lipomatosis complicated by intussusception in an adult: A case report	International Journal of Surgery Case Reports	47	M	Abdominal pain	5 days	Ileum	3	Y	Laparotomy	5
**Kuzmich et al.**	2010	Ileocolocolic intussusception secondary to a submucosal lipoma: an unusual cause of intermittent abdominal pain in a 62-year-old woman	Journal of Clinical Ultrasound	62	F	Abdominal pain, weight loss	2 months	Ileum	7	Y	Laparotomy	Not stated
**Lee et al.**	2010	A case of small-bowel intussusception caused by intestinal lipomatosis: preoperative diagnosis and reduction of intussusception with double-balloon enteroscopy	Gastrointestinal Endoscopy	48	F	Abdominal pain, weight loss	2 months	Jejunum	5	Y	Laparoscopic	Not stated
**Lee et al.**	2017	Ileocolic intussusception caused by a lipoma in an adult	World Journal of Clinical Cases	29	F	Abdominal pain, nausea, fever	1 day	Ileum	3.5	Y	Laparoscopic	8
**Li et al.**	2018	Gastrointestinal hemorrhage caused by adult intussusception secondary to small intestinal tumors: Two case reports	Medicine (Baltimore)	54	M	GI bleed	1 day	Ileum	5	Y	Laparoscopic	5
**Lill et al.**	2007	Multiple lipomatosis - A rare cause for small bowel intussusception	New Zealand Medical Journal	39	M	Abdominal pain	3 months	Jejunum	3	Y	Laparotomy	Not stated
**Lin et al.**	2007	Laparoscopy-assisted resection of ileoileal intussusception caused by intestinal lipoma	Journal of Laparoendoscopic and Advanced Surgical Techniques	31	M	Anaemia, GI bleed	1 year	Ileum	4	Y	Laparotomy	8
**Lucas et al.**	2011	Laparoscopic resection of a small bowel lipoma with incidental intussusception	Journal of the Society of Laparoendoscopic Surgeons	73	M	Anaemia, GI bleed	Not stated	Jejunum	2.1	Y	Laparoscopic	3
**Manna et al.**	2017	A rare cause of acute gastrointestinal hemorrhage: ileal lipoma Case report	Italian Annals of Surgery	66	M	Anaemia, GI bleed	Not stated	Ileum	Not stated	Y	Laparotomy	Not stated
**Manouras et al.**	2007	Lipoma induced jejunojejunal intussusception	World Journal of Gastroenterology	55	M	Abdominal pain, GI bleed	3 months	Jejunum	4	Y	Laparotomy	7
**Mazziotti et al.**	2006	Macrodactylia fibrolipomatosis associated with multiple small-bowel lipomas	American Journal of Roentgenology	57	M	Abdominal pain, diarrhoea	10 years	Jejunum	4	N	Not stated	Not stated
**Mbaye et al.**	2017	[Volvulus of the small intestine caused by mesenteric lipoma]	Pan African Medical Journal	7	F	Abdominal pain, vomiting	6 days	Ileum	Not stated	Y	Laparotomy	Not stated
**McCoubrey et al.**	2006	Small bowel volvulus secondary to a mesenteric lipoma: A case report and review of the literature	Irish Journal of Medical Science	40	M	Abdominal pain, vomiting, constipation	7 days	Mesentery	16	Y	Laparotomy	8
**McKay**	2006	Ileocecal intussusception in an adult: the laparoscopic approach	Journal of the Society of Laparoendoscopic Surgeons	63	M	Abdominal pain, nausea, GI bleed	1 day	Ileoceacal valve	Not stated	Y	Laparotomy	5
**Meshikhes et al.**	2005	Adult intussusception caused by a lipoma in the small bowel: report of a case	Surgery Today	55	M	Abdominal pain, nausea, abdominal distension		Ileum	6	Y	Laparotomy	5
**Minaya Bravo et al.**	2012	Ileocolic intussusception due to giant ileal lipoma: Review of literature and report of a case	International Journal of Surgery Case Reports	75	M	Abdominal pain, diarrhoea, vomiting	3 months	Ileum	5.5	Y	Laparotomy	9
**Morimoto et al.**	2010	Peeling a giant ileal lipoma with endoscopic unroofing and submucosal dissection	World Journal of Gastroenterology	62	M	GI bleed	Not stated	Ileum	5	Y	Endoscopic	7
**Mouaqit et al.**	2012	Adult intussusceptions caused by a lipoma in the jejunum: report of a case and review of the literature	World Journal of Emergency Surgery	35	M	Abdominal pain, nausea	4 months	Jejunum	6	y	Laparotomy	Not stated
**Nakanishi et al.**	2019	Laparoscopic-endoscopic cooperative surgery for ileal lipoma: A case report	Asian Journal of Endoscopic Surgery	50	M	GI bleed	Not stated	Ileum	2.5	N	Laparoscopic	Not stated
**Noda et al.**	2016	Successful endoscopic submucosal dissection of a large terminal ileal lipoma	Case Reports in Gastroenterology	78	F	Abdominal pain	1 year	Ileum	3	N	Endoscopic	Not stated
**Ooi et al.**	2015	Bleeding ileal lipoma: An extremely rare presentation of anemia in adults	Journal of Gastroenterology and Hepatology (Australia)	27	M	GI bleed	1 Week	Ileum	Not stated	Y	Laparotomy	Not stated
**Oyen et al.**	2007	Ileo-ileal intussusception secondary to a lipoma: A literature review	Belgian Acta Chirurgica Belgica	54	M	Not stated	Not stated	Ileum	Not stated	Y	Laparoscopic	Not stated
**Pandya et al.**	2013	Laparoscopic management of intussusception in an adult	Surgical Endoscopy and Other Interventional Techniques	47	F	Abdominal pain	1 Month	Not stated	3	Y	Laparoscopic	Not stated
**Papageorge et al.**	2018	Pedunculated small bowel lipoma with heterotopic pancreas causing intussusception	Clinical Case Reports	36	M	Abdominal pain, abdominal distension	4 months	Ileum	6.5	Y	Laparoscopic	Not stated
**Parmar et al.**	2004	Submucous lipoma of the ileocaecal valve presenting as cecal volvulus	International Journal of Clinical Practice	53	F	Abdominal pain	1 Day	Ileocaecal valve	Not stated	y	Laparotomy	Not stated
**Paya Llorente et al.**	2018	Laparoscopic surgery for adult enterocolic intussusception: Case report and literature review	Gastroenterology Hepatology	20	M	Abdominal pain, GI bleed	1 Day	Ileocaecal valve	4.3	Y	Laparoscopic	7
**Pezzoli et al.**	2008	Occult intestinal hemorrhage due to lipoma of the small bowel detected with the combined use of the new endoscopic techniques. A report of two cases	Digestive and Liver Disease	64	M	Anaemia	Not stated	Jejunum	4	Y	Not stated	Not stated
**Rathore et al.**	2006	Adult intussusception--a surgical dilemma	Journal of Ayub Medical College, Abbottabad	65	F	Abdominal pain, GI bleed	8 months	Ileocaecal valve	Not stated	Y	Laparotomy	Not stated
				60	F	Obstruction	Few days	Ileum	Not stated	Y	Laparotomy	Not stated
**Rattan et al.**	2013	Small bowel lipomas may be a cause of significant obscure GI bleeding: Report of three cases identified by capsule endoscopy	Journal of Gastroenterology and Hepatology	66	M	Abdominal pain, GI bleed, anaemia	Not stated	Jejunum	2.8	Y	Laparoscopic	Not stated
				76	F	GI bleed	Not stated	Jejunum	Not stated	Y	Not stated	Not stated
**Ross et al.**	2000	Case 26: Jejunojejunal intussusception secondary to a lipoma	Radiology	80	M	Abdominal pain	Not stated	Jejunum	Not stated	Y	Not stated	Not stated
**Safatle-Ribeiro et al.**	2016	Obscure gastrointestinal bleeding caused by intestinal lipomatosis: double-balloon endoscopic and laparoscopic views	Endoscopy	52	M	Abdominal pain, GI bleed	6 years	Jejunum	Not stated	Y	Laparoscopic	3
**Saito et al.**	2013	Laparoscopy-assisted resection of ileocecal intussusception caused by ileal pedunculated lipoma	International Journal of Surgery	31	M	GI bleed, anaemia	1 year	Ileum	4	Y	Laparoscopic	8
**Seow-En et al.**	2014	Jejunojejunal intussusception secondary to submucosal lipoma resulting in a 5-year history of intermittent abdominal pain	British Medical Journal Case Reports	44	F	Abdominal pain, nausea	5 years	Jejunum	3	Y	Laparoscopic	Not stated
**Shah et al.**	2005	Mesenteric lipoma leading to small gut strangulation and short syndrome	Journal of the College of Physicians and Surgeons Pakistan	14	M	Abdominal pain, vomiting	1 day	Jejunum	12	Y	Laparotomy	Not stated
**Sheehan et al.**	2000	Intussusception in adults: A rare entity	Irish Journal of Medical Science	53	M	Abdominal distention, vomiting, diarrhoea.	1 Week	Ileum	Not stated	Y	Laparotomy	Not stated
**Sheen et al.**	2003	A small bowel volvulus caused by a mesenteric lipoma: Report of a case	Surgery Today	31	M	Abdominal pain, nausea, vomiting	2 days	Ileum	10	Y	Laparotomy	Not stated
**Shenoy et al.**	2003	Segmental jejunal lipomatosis - A rare cause of intestinal obstruction	Yonsei Medical Journal	33	M	Abdominal pain, abdominal distension	Not stated	Jejunum	Not stated	Y	Laparotomy	Not stated
**Shiba et al.**	2009	Preoperative Diagnosis of Adult Intussusception Caused by Small Bowel Lipoma	Case Reports in Gastroenterology	33	M	Abdominal pain	2 weeks	Ileum	4	Y	Laparotomy	Not stated
**Shimazaki et al.**	2015	Laparoscopic management of an octogenarian adult intussusception caused by an ileal lipoma suspected preoperatively: A case report	World Journal of Surgical Oncology	87	M	Abdominal distention, vomiting	2 weeks	Ileum	4	Y	Laparoscopic	8
**Singh et al.**	2013	Intussusception due to jejunal lipoma: A case report	Journal of International Medical Sciences Academy	22	M	Abdominal pain, nausea, vomiting	5 days	Jejunum	6	Y	Laparotomy	5
**Spada et al.**	2013	Giant Lipoma as an Unusual Cause of Obscure Gastrointestinal Bleeding	Video Journal and Encyclopedia of GI Endoscopy	62	M	GI bleed	Not stated	Ileum	3.6	Y	Laparoscopic	Not stated
**Spaventa-Ibarrola et al.**	2006	Ileocecal valve lipoma. Case report and review of the literature	Spanish Surgery and Surgeons	78	F	Obstruction, abdominal distention, constipation	Not stated	Ileum	2.5	Y	Laparotomy	Not stated
**Stancu et al.**	2016	Ileo-colic intussusception by ileo-cecal valve lipoma - an infrequent ultrasonographic occurrence. A case report	Journal of Medical Ultrasound	52	F	Abdominal pain, weight loss, constipation	1 month	Ileum	5.5	Y	Laparotomy	Not stated
**Suairez Moreno et al.**	2010	Multiple intestinal lipomatosis. Case report	Spanish Surgery and Surgeons	51	M	Abdominal pain, nausea	Not stated	Multiple	Not stated	N	Conservative	Not stated
**Suga et al.**	2019	Giant Mesenteric Lipoma Causing Small Bowel Volvulus: A Case Report	Ethiopian Journal of Health Sciences	25	M	Abdominal pain	3 days	Jejunum	15	Y	Laparotomy	Not stated
**Tayeh et al.**	2015	Giant mesenteric lipoma: A case report and a review of the literature	Journal of Pediatric Surgery Case Reports	2	M	Abdominal distension	1 year	Ileum	22	Y	Laparotomy	Not stated
**Toya et al.**	2014	Lipoma of the small intestine treated with endoscopic resection	Clinical Journal of Gastroenterology	79	M	GI bleed	Not stated	Jejunum	3.5	Y	Endoscopic	Not stated
**Tse et al.**	2018	Intermittent intussusception and microcytic anemia caused by a submucosal jejunal lipoma: A rare case report	Surgical Endoscopy and Other Interventional Techniques	40	M	Abdominal pain, anaemia, GI bleed	3 weeks	Jejunum	5.5	Y	Laparoscopic	2
**Tsushimi et al.**	2006	Laparoscopic resection of an ileal lipoma: Report of a case	Surgery Today	63	F	Abdominal pain, vomiting	Not stated	Ileum	2.5	N	Laparoscopic	15
**Turi et al.**	2004	Lipoma of the Small Bowel - A Rare Cause of Abdominal Pain and Chronic Bloody Diarrhoea	German Journal of Gastroenterology	40	F	Abdominal pain, diarrhoea, weight loss	6 weeks	Ileum	2	Y	Not stated	Not stated
**Uyulmaz et al.**	2018	Ileoileal intussusception in unspecific recurrent abdominal pain in adult: A case report	SAGE Open Medical Case Reports	53	F	Abdominal pain, diarrhoea, weight loss	3 months	Ileum	8	Y	Laparoscopic	8
**Vagholkar et al.**	2015	Lipoma of the Small Intestine: A Cause for Intussusception in Adults	Case Reports in Surgery	22	M	Abdominal pain, vomiting	2 days	Ileum	Not stated	Y	Laparotomy	Not stated
**Vekic et al.**	2014	Pedunculated obstructive lipoma of the ileocecal valve: a case report	Serbian Archives of Medicine	67	F	Abdominal pain, nausea, abdominal distension, vomiting, constipation	3 days	Ileocaecal valve	5	Y	Laparotomy	7
**Wan et al.**	2010	Partial intestinal obstruction secondary to multiple lipomas within jejunal duplication cyst: A case report	World Journal of Gastroenterology	68	M	Abdominal distention, weight loss	10 days	Jejunum	3.2	Y	Laparotomy	7
**Wardi et al.**	2013	Unusual cause of upper gastrointestinal bleeding	Journal of Medical Case Reports	53	M	GI bleed, anaemia	6 months	Jejunum	5	Y	Laparotomy	Not stated
**Watt et al.**	2012	Mesenteric lipoma causing small bowel perforation: A case report and review of literature	Scottish Medical Journal	72	M	Abdominal pain	Not stated	Ileum	Not stated	Y	Laparotomy	Not stated
**Wolko et al.**	2003	Torsion of a giant mesenteric lipoma	Pediatric Radiology	9	M	Abdominal pain	10 days	Ileum	Not stated	Y	Laparotomy	Not stated
**Wu et al.**	2018	Preoperative radiologic patent blue localization for intracorporeal laparoscopic resection of a terminal ileal submucosal lipoma: A case report	International Journal of Surgery Case Reports	31	F	Abdominal pain	Not stated	Ileum	1.5	Y	Laparoscopic	Not stated
**Yagnik**	2018	Giant ileocecal submucosal lipoma presenting with hematochezia	ANZ Journal of Surgery	65	M	GI bleed	15 Days	Ileocaecal valve	5	Y	Laparotomy	Not stated
**Yatagai et al.**	2016	Obscure gastrointestinal bleeding caused by small intestinal lipoma: a case report	Journal of Medical Case Reports	69	M	GI bleed, anaemia	Not stated	Jejunum	3.6	Y	Laparoscopic	9
**Yigitler et al.**	2007	A rare cause of bleeding intestinal intussusception in adult: jejunal lipoma	Turkish Journal of Trauma and Emergency Surgery	76	M	Obstruction, GI bleed	Not stated	Jejunum	Not stated	N	Conservative	Not stated
**Yoshimoto et al.**	2019	Novel surgical approach without bowel resection for multiple gastrointestinal lipomatosis: A case report	International Journal of Surgery Case Reports	47	F	Obstruction	Not stated	Ileum	4.3	Y	Laparoscopic	Not stated
**Zissin**	2004	Enteroenteric intussusception secondary to a lipoma: CT diagnosis	Emergency Radiology	20	F	Abdominal pain, vomiting	1 month	Ileum	1.8	Y	Laparotomy	Not stated
**Gray et al.**	2001	Small intestinal intussusception secondary to a submucosal lipoma	Archives of pathology and laboratory medicine	64	F	Abdominal pain	9–12 months	Not stated	3.5	Y	Laparotomy	Not stated
**Balamoun et al.**	2011	Ileal lipoma-a rare cause of ileocolic intussusception in adults: case report and literature review	World Journal of Gastroenterology Surgery	65	M	Abdominal pain, vomiting	3 days	Ileum	1.2	Y	Laparotomy	Not stated
**Colovic et al.**	2000	Mesenteric lipoma causing volvulus of the small intestine	Serbian Archives of Medicine	77	M	Abdominal pain, vomiting	5 days	Mesentery	18	Y	Laparotomy	Not stated
**Wong et al.**	2005	Primary mesenteric lipoma causing closed loop bowel obstruction: a case report	The Kaoshiung Journal of Medical Sciences	45	F	Abdominal pain	Sudden onset	Ileum	6.5	Y	Laparotomy	Not stated
**Aminian et al.**	2009	Ileal intussusception secondary to both lipoma and angiolipoma: a case report	Cases Journal	53	F	Abdominal pain, nausea, diarrhoea	4 months	Ileum	1.5	Y	Laparotomy	7
**Lin et al.**	2007	Laparoscopy-assisted resection of ileoileal intussusception caused by intestinal lipoma	Journal of Laparoendoscopic and Advanced Surgical Techniques	47	F	Abdominal pain, nausea, vomiting	5 days	Ileum	3	Y	Laparotomy	4
**Zografos et al.**	2005	Small intestinal lipoma as a cause of massive gastrointestinal bleeding identified by intraoperative enteroscopy. A case report and review of the literature	Digestive diseases and Sciences	82	F	GI bleed	2 days	Ileum	2.5	Y	Laparotomy	9
**Park et al.**	2001	Laparoscopic-assisted resection of ileal lipoma causing ileo-ileo-colic intussusception	Journal of Korean Medical Sciences	39	M	Abdominal pain	2 years	Ileum	4	Y	Laparoscopic	4
**Cha et al.**	2009	Giant mesenteric lipoma as an unusual cause of abdominal pain: a case report and a review of the literature	Journal of Korean Medical Sciences	29	F	Abdominal pain, abdominal distension, constipation	3 years	Mesentery	19	Y	Laparoscopic	Not stated
**Charalambous et al.**	2012	Jejunojejunal lipoma causing intussusception	Case Reports in Gastroenterology	46	M	Abdominal pain, GI bleed	3 months	Jejunum	4	Y	Laparotomy	7
**Jai et al.**	2008	Jejunal lipoma with intermittent intussusception revealed by partial obstructive syndrome	The Saudi Journal of Gastroenterology	37	F	Abdominal pain	3 years	Jejunum	3	Y	Laparotomy	Not stated
**Chen et al.**	2008	Severe adult ileosigmoid intussusception prolapsing from the rectum: A case report	Cases Journal	36	M	Abdominal pain, diarrhoea and rectal prolapse	2 months	Ileocaecal valve	9	Y	Laparotomy	Not stated
**Enyinnah et al.**	2013	Mesenteric lipoma causing recurrent intestinal obstruction	Nigerian Journal of Clinical Practice	29	M	Abdominal pain, vomiting, constipation, abdominal mass	10 years	Mesentery	15	Y	Laparotomy	Not stated
**Innocent et al.**	2015	Distal ileal stenosing subserosal lipoma: a case report	Nigerian Journal of Medicine	38	M	Obstruction	Not stated	Ileum	Not stated	Y	Laparotomy	7
**Jiang et al.**	2015	Pancreatic and Gastric Heterotopia with Associated Submucosal Lipoma Presenting as a 7-cm Obstructive Tumor of the Ileum: Resection with Double Balloon Enteroscopy	Case Reports in Gastroenterology	38	F	Abdominal pain, nausea, vomiting, GI bleed	29 years	Ileum	12.5	Y	Endoscopic	Not stated
**Kabawe et al.**	2019	Jejunal intussusception in an adult due to multiple lipomas: a rare case report from Syria	Journal of Surgical Case Reports	37	M	Abdominal pain, vomiting, abdominal distension	3 days	Jejunum	4.5	Y	Laparoscopic	3
**Lee et al.**	2013	Endoscopic treatment of a symptomatic ileal lipoma with recurrent ileocolic intussusceptions by using cap-assisted colonoscopy	Clinical Endoscopy	73	F	Abdominal pain, weight loss	2 years	Ileum	2.7	Y	Endoscopic	Not stated
**Molnar et al.**	2013	Ileo-ceco-descendento-colic intussusception in adult - a case report	Romanian Journal of Surgery	30	F	Abdominal pain, nausea, vomiting, weight loss	10 days	Ileum	5	Y	Laparotomy	7
**Namikawa et al.**	2011	Adult ileoileal intussusception induced by an ileal lipoma diagnosed preoperatively: report of a case and review of the literature	Surgery Today	68	F	Abdominal pain	Not stated	Ileum	1.5	Y	Laparotomy	10
**Pinto et al.**	2018	Jejunal Lipoma, an Uncommon Cause of Gastrointestinal Bleeding	Portuguese Journal of Gastroenterology	46	M	GI bleed, fatigue	Not stated	Jejunum	7.5	Y	Laparotomy	Not stated
**Shpaner et al.**	2008	Rectal bleeding caused by a large, partially obstructing lipoma of the terminal ileum	Clinical Gastroenterology and Hepatology	38	F	GI bleed, weight loss	2 months	Ileum	3.3	Y	Laparoscopic	Not stated
**Sueoka et al.**	2016	A Case of Spontaneously Reduced Ileoileal Intussusception Caused by a Lipoma	Hiroshima Journal of Medical Sciences	68	F	Abdominal pain	Sudden onset	Ileum	2.5	Y	Laparotomy	Not stated
**Yang et al.**	2017	Torsion of a Giant Antimesenteric Lipoma of the Ileum: A Rare Cause of Acute Abdominal Pain	The American Journal of Case Reports	67	F	Abdominal pain, abdominal distension, nausea, vomiting	1 week	Ileum	12	Y	Laparotomy	7

GI = Gastrointestinal, M = Male, F = Female, Y = Yes, N = No.

Presenting symptoms were reported as (Table 2): abdominal pain 101 (68.7%); nausea/vomiting 52 (35.3%), hematochezia/GI bleeding 49 (33.3%); anaemia 16 (10.9%); abdominal distension 18 (12.2%); constipation 13 (8.9%); weight loss 11 (7.5%); small bowel obstruction 7 (4.7%); diarrhoea 6 (4.1%); fatigue 4 (2.7%); fever 2 (1.4%); symptoms not specified 2 (1.4%).

**Table 2:**
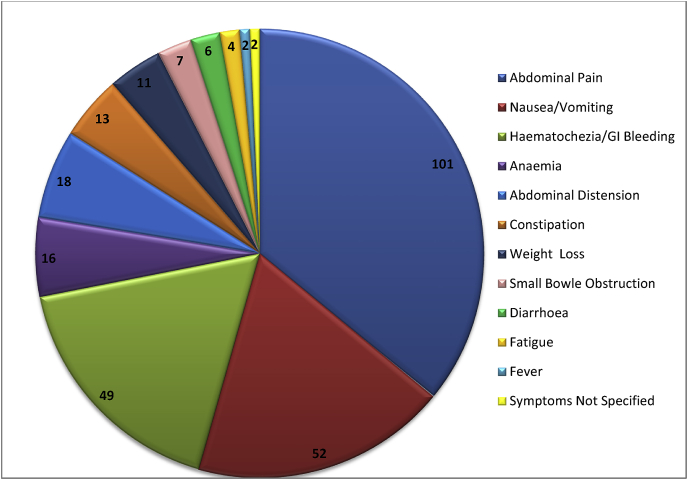
Presenting symptom profile

All patients were symptomatic and 134/147 (91.2%) presented as emergencies. Duration of preceding symptoms varied from 1 h to 29 years and was recorded in 104/147 cases. Mean duration of preceding symptoms was 295 days and standard deviation (SD) = 1173. With symptoms greater than 1 year excluded (n = 9), mean preceding symptom length was 58.1 days (SD = 96.8).

Diagnostic imaging modalities were: abdominal X-Ray 49/147 (33.3%); endoscopy 68/147 (46.3%); CT 115/147 (78.2%); abdominal ultrasound 35/147 (23.8%); barium study 20/147 (13.6%); video capsule endoscopy (VCE) 13/147 (8.8%); MRI small bowel 4/147 (2.7%).

124/137 (90.5%) required definitive surgical management, either laparotomy (n = 89) or laparoscopic resection (n = 35). 8 laparotomies started as laparoscopic procedures and 1 as an attempted endoscopic resection. 13 patients were successfully managed non-operatively (9.5%); 9 with endoscopic resection (6.6%) and 4 conservatively (2.9%). In 10 cases the definitive management strategy was not stated (see Table 3).

**Table 3:** Comparison of management strategies.

	Laparotomy	Laparoscopic	Endoscopic	Conservative	Not stated
**Initial Management**	80	43	10	4	10
**Definitive Management**	89	35	9	4	10
**Success (%)**	NA	81%	90%	100%	NA

**Fig. 2:**
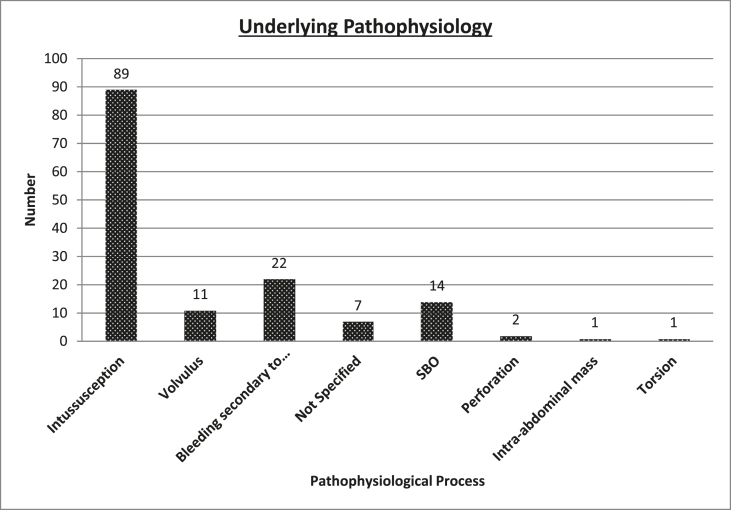
Underlying pathophysiology.

The underlying pathophysiology was intussusception 89 (60.5%); bleeding secondary to ulceration/necrosis 22 (15%); volvulus 11 (7.5%), small bowel obstruction 14 (9.5%); perforation 2 (1.4%); intra-abdominal mass 1 (0.7%); torsion 1 (0.7%) not specified 7 (4.8%). Fig. 2 highlights these results.

Location of lipoma was ileum (n = 88, 59.9%); jejunum (n = 47,32%); mesentery (n = 7, 4.8%); multiple (n-2, 1%) not specified (n = 3, 2%). The ileal cases can be further subdivided: ileum (n = 75; ileocaecal valve (n = 10); Meckel's diverticulum (n = 3).

Lipoma size was recorded in 115 cases and ranged from 1.2 to 22 cm at the greatest diameter. Mean size was 5.1 cm. When comparing mean lipoma size and successful management strategy, laparotomy = 5.6 cm, laparoscopic = 4.4 cm, endoscopically managed 3.7 cm, conservative 4.5 cm (Table 4. One-way Anova test was performed, the p value of 0.21 demonstrated no statistically significant difference between groups (laparotomy, laparoscopic and endoscopic).

**Table 4:** Associations between management, lipoma size and length of stay.

Definitive Management	Number	Average Size (cm)	Length of stay (days)
Laparotomy	89	5.6	8.5
Laparoscopic	35	4.4	6.4
Endoscopic	9	3.7	7
Conservative	4	4.5	not stated
Not stated	10	/	/
Overall	147	5.1	7.4

Of the 147 cases, one report described the specimen as a chondrolipoma. All other cases were benign lipomas.

**Fig. 3:**
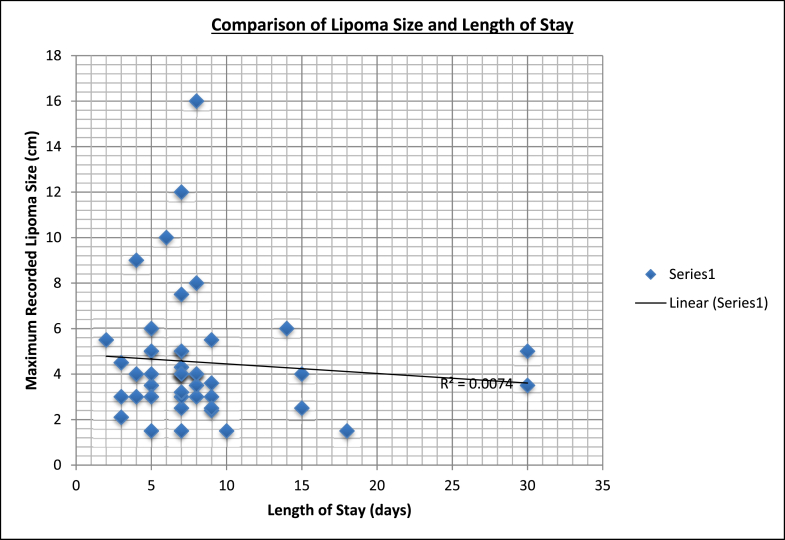
Comparison of lipoma size and length of stay.

Average length of hospital stay (n = 68) was 7.4 (2–30) days. Interquartile range = 3 (Q3–Q1 (8-5)). Average length of stay was 8.5 days with open surgery and 6.4 days with laparoscopic surgery (Table 4). T-test was performed, analysing length of stay between laparotomy and laparoscopic management. A p value of 0.13 demonstrated no statistically significant difference in length of stay. Numbers did not permit comparison of length of stay with the other management modalities. Comparison of lipoma size and length of stay in the 52 cases where both variables were recorded is shown in Fig. 3. There was no significant correlation (R^2^ = 0.0074).

4 complications were reported from the 135 cases: 2 surgical wound infections; multi-organ failure and PE; intraoperative laceration to muscular layer. No associated intraoperative or 30-day mortality was reported.

## 4 Discussion

Our data identify a male preponderance (60%) in those with symptomatic small bowel lipomas. Lipomas of the colon are reported as being more common in women [8], whereas those found in the oesophagus have a greater prevalence in men [9]. Gastrointestinal lipomas are most commonly found in patients aged 50–70 years [10,11]. Average patient age of this cohort (49.9 years) lies just outside this range (however this is comparable with other reported groups of patients with lipomas). Our data emphasise that lipomas may present at any age with both children and the elderly documented. Pediatric gastrointestinal lipomas are rarely encountered. Our study highlights 9 pediatric cases of symptomatic lipomas causing intussusception, volvulus, abdominal mass or obstruction.

Lipomas of the gastrointestinal tract have been extensively documented as causative factors in bleeding, intussusception, obstruction, volvulus and altered bowel habit. There is wide variation in presentation. (Our data highlights the breadth of presenting symptoms.) Abdominal pain was the most prevalent symptom, reported in 68% of patients, whilst nausea and/or vomiting and gastrointestinal bleeding were also commonly seen, in 35% and 34% of patients respectively. This is not surprising given that 60% of cases were related to intussusception.

Our results are consistent with data from other papers which identify chronic intermittent cramping abdominal pain associated with nonspecific signs of bowel obstruction including nausea, vomiting, gastrointestinal bleeding, constipation or abdominal distension as key symptoms associated with intussusception [12]. Lipomas accounts for 5% of all cases of intussusception in adults [13], the rest of which are mainly caused by malignant neoplasm [14].

The time course of presenting symptoms ranged from only a few hours to many years. Whilst there was considerable discrepancy in time course within our data, the mean of 58.1 days of preceding symptoms (when 9 results >1 year were excluded) serves as an indicator as to the most commonly encountered presentation. The wide variation may be explained by the fact that many patients had undergone semi-urgent/elective diagnostic investigations in the community prior to presenting as an emergency.

Gastrointestinal endoscopic investigations are viewed as the gold standard to investigate red flag symptoms of malignancy, bleeding, weight loss, on-going abdominal pain and anaemia [15]. Such symptoms are common to both gastrointestinal malignancy and symptomatic lipomas. However, endoscopic investigations are often negative in lipoma patients given the anatomical position of small bowel lipomas. Thus, delay in diagnosis and referral on for further investigations are likely outcomes.

It is not surprising that the majority of patients in our cohort underwent numerous investigations prior to definitive diagnosis and management. Negative endoscopic investigations were a recurrent theme in many. Given documented colonoscopy perforation rates of 0.016%–0.2% [16], are these patients being exposed to unnecessary risk of potential morbidity? This is pertinent, as radiological imaging is an effective diagnostic tool for lipomas. Nevertheless, malignancy is a differential diagnosis and CT alone may miss a small bowel cancer, diagnosis is only accurate in 55% of cases [17]. Thus, endoscopic work up is an important adjunct helping clinicians exclude other more common pathology despite associated risks.

As stated conventional endoscopic investigations such as colonoscopy and gastroscopy are negative in this cohort. However, double balloon enteroscopy (DBE) appears both an effective diagnostic and therapeutic modality enabling direct visualisation, biopsy and resection of small bowel lipomas in the appropriate setting. The ability to offer therapeutic treatment sets this option out from other diagnostic modalities such as video capsule endoscopy [18]. Nevertheless, DBE is an invasive procedure and is limited to specialist centres. Currently DBE does not appear to form part of standard diagnostic work in this patient cohort.

The sensitivity and specificity of ultrasound in the diagnosis of lipomas are reported as being 85.71% and 95.95% respectively by Rahmani et al. [19]. However, transabdominal ultrasonography is not accurate for detecting small bowel tumors; the reported sensitivity is low (26%) [20]. In contrast, CT and MRI both have high sensitivity in detecting gastrointestinal lipomas [21]. It therefore follows that the majority of patients underwent CT imaging (78%).

More lipomas were located in the ileum than jejunum (59.9%–32% respectively). Our data support previous reports that ileal lipomas are more prevalent than jejunal lipomas [22,23].

Manouras et al. state ‘lesions less than 1 cm are considered incapable of producing symptoms, while 75% of those greater than 4 cm are symptomatic’ [4]. Our data support this statement, with the average maximal diameter in symptomatic lipomas measuring 5.1 cm. No lipoma less than 1.2 cm was recorded within our dataset. When evaluating whether any association between maximum lipoma diameter and successful treatment modality exists our results suggest that larger lipomas are more likely to undergo surgery (surgically managed = 5.1 cm, endoscopically managed 3.7 cm, conservative 4.5 cm). Caution when interpreting such results should be taken given the small sample sizes of those managed conservatively and endoscopically.

No reports of surveillance relating to small bowel lipoma growth are reported. One may postulate that even incidentally found large (>2 cm) asymptomatic small bowel lipomas do not require routine follow up given the rarity of patients becoming symptomatic and very low associated risk of malignant transformation.

Various pathophysiological mechanisms are shown in Fig. 2. Some are related, with gastrointestinal bleeding occurring as a result of pressure necrosis and ulceration, and obstruction when a lipoma occludes the bowel lumen. Intussusception and volvulus are similarly capable of causing obstruction and bleeding. Intussusception was the most common pathophysiological mechanism within our cohort. Our data give an up to date review of ways in which lipomas give rise to pathology in these patients.

With few documented cases, no consensus on the management of symptomatic small bowel lipomas currently exists. Parallels can be drawn from the management of colonic lipomas where Nallamothu et al. advocate surgery as first line treatment in lipomas that are sessile, with limited peduncles, extension into muscularis propria/serosa, or when endoscopic resection has failed [8]. Surgery is also suggested as primary management for giant colonic lipomas (>4 cm). However, we suggest other strategies may sometimes have a role.

Conservative management alone was effective in 4 patients. Spontaneous expulsion of a 7 × 4.5 × 3.6 cm ileal lipoma resolved a patient's intussusception and negated the need for surgical intervention as described by Kang [24]. Kim et al. report a 50-year-old man who declined surgery after double balloon enteroscopy diagnosed multiple jejunal lipomatosis [25]. He was treated with analgesia and followed up regularly as an outpatient. Suarez et al. document a 51-year-old male found to have multiple submucosal lipomas in the stomach and small bowel [26]. His symptoms spontaneously settled without the need for any treatment. Nevertheless, these cases appear to be the exception within this cohort.

Endoscopic mucosal resection (as part of DBE) appears to have a limited role in the management of small bowel lipomas. Given the anatomical constraints and required expertise of such procedures this practice is not widespread and accounts for only a small portion of those managed. Nevertheless, successful procedures have been undertaken, as evidenced by our data. Noda et al. report endoscopic mucosal dissection of a 3 cm terminal ileal lipoma [27], whilst Morimoto used a combination of endoscopic snare and IT-knife to perform endomucosal dissection of a 5 cm ileal lipoma although this was complicated by a muscular and serosal layer laceration [28]. Javia reports a patient with a 2 cm terminal ileal lipoma which was excised using endoscopic snare [29]. A patient with a 2 cm lipoma underwent double-balloon-assisted jejunal endoscopic mucosal resection, as reported by Kröner et al. [30]. Such reports demonstrate that both jejunal and ileal lipomas may be managed by endoscopic measures. Only one reported case failed to remove the lipoma, citing the size (3 × 1.5 × 1.5 cm) and wide base as reasons for this. A subsequent laparotomy was required to treat the patient [31]. Careful case selection appears to be an important factor, with some authors stating risks of bleeding and perforation as contraindications to undertaking such procedures [32]. Of the 10 attempted endomucosal resections, 9 were published from 2012 onwards, indicating that this is an emerging area within endoscopy.

Our results show that surgery was the most utilised definitive management strategy. Both open and laparoscopic procedures were undertaken with preponderance for laparotomy as definitive management. Those patients requiring surgery primarily underwent bowel resection and primary anastomosis. Anatomical location determined whether resection was only small bowel or included a portion of large bowel. As Table 4 demonstrates, the average size of symptomatic lipoma resected laparoscopically was (1.2 cm) smaller than those removed via open surgery, however, the exact reasons for this is unclear. Patient selection is likely to a be a factor, with multiple aspects taken into consideration e.g. a surgeon's skillset/standard practice, a centres equipment, critical condition of a patient, degree of bowel obstruction, patient comorbidities and lipoma size. The high rate of surgical management may be attributable to the need to exclude alternative causes for each presentation such as malignancy and the limited practice of alternative management strategies [14,33].

Laparoscopic surgery was unsuccessful in 19% of cases attempted. Authors state a variety of reasons for converting to open surgery. Alsayegh et al. report the use of laparoscopy being diagnostic in a 4 year old before converting to a Pfannenstiel incision in order to resect a 6.7 × 7.6 × 4.4 cm lipoma of the mesentery causing volvulus [34]. Bilgin states that intraoperative adhesions in a case of adult intussusception secondary to a lipoma resulted in conversion [35]. The cost of laparoscopic staplers is highlighted as a factor by Lin for performing a laparoscopy-assisted extracorporeal resection and anastomosis of an intussuscepted segment [36]. Sheehan cites oedema and ischaemia following attempted laparoscopic reduction of an ileocolic intussusception [37].

Associated mortality (0%) and morbidity (2%) rates were low. Given that over 90% of patients underwent surgical intervention in a cohort where average age was almost 50 years, such values are encouraging. However, comparison of morbidity and mortality associated with similar pathologies suggests that complications may have been underreported or not documented. Mortality from adult intussusception increases from 8.7% for benign lesions to 52.4% for those with a malignant cause [38]. Although there are numerous documented cases of gastrointestinal lipomas associated with intussusception, very few report associated morbidity.

Crocetti et al. report an average length of stay in hospital of 5 and 7 days in patients with symptomatic colonic lipomas managed laparoscopically and with open surgery respectively [39]. In our cohort the average length of stay with symptomatic small bowel lipomas was 7.4 days. Open surgery was associated with a longer length of stay (8.5 days) when compared to laparoscopic management (6.4 days). These results are consistent with other reports of shorter hospital stays with laparoscopic management of small bowel obstruction [40].

We acknowledge that there are limitations associated with our study. The paper is based on only those cases documented in the literature. The true incidence of symptomatic lipomas is likely to be higher. Equally we take into account reporting bias. Sub-acute symptomatic patients are unlikely to require emergency intervention and equally will not be reported on. Nevertheless, based on the data collated and analysed we feel able to draw rational conclusions.

## 5 Conclusion

We provide a topical and current overview of symptomatic small bowel lipomas. Numerous reports exist of individual cases, referencing small observational studies dating back many decades, but little new data concerning this relatively unknown condition has been collated in recent years. Our study is up to date and practical, presenting new findings, helping provide a framework for classification and management.

A number of important and previously undocumented points are illustrated. A clearer symptom profile is described with most presenting as emergencies necessitating tailored patient care in a timely fashion. Computerised tomography appears to be the primary diagnostic investigation, helping delineate both lipoma and sequelae. Lipomas >1.2 cm may be symptomatic although larger lipomas appear more implicated. Open surgery remains the primary management modality, but smaller symptomatic lipomas may be targeted for laparoscopic surgery in appropriate settings. Laparoscopic surgery is associated with shorter hospitals stays. Endoscopic resection may be a practical first line management in carefully selected patients, although limited data currently exist. Such techniques may reduce the need for invasive surgery in future as skillset and availability improve. Morbidity and mortality rates appear low in this cohort of patients irrespective of lipoma size or management strategy.

We hope that this study offers an insight into the many different facets associated with symptomatic small bowel lipomas. This study adds to the scanty existing knowledge about symptomatic small bowel lipomas. It will inform clinicians and guide management in both the elective and emergency setting to help achieveoptimal patient outcomes.

## Provenance and peer review

Not commissioned externally peer reviewed.

## Funding

None.

## Ethical approval

Not Applicable.

## Consent

Not applicable.

## Registration of research studies

1. Name of the registry: PROSPERO.

2. Unique Identifying number or registration ID: CRD42020172916.

3.Hyperlink to your specific registration (must be publicly accessible and will be checked): https://www.crd.york.ac.uk/PROSPERO/display_record.php?RecordID=172916

## Author contribution

Nicholas Farkas : study design, data collection, data analysis, writing, editing

Joshua Wong : data collection, data analysis

Jordan Bethel : Data collection

Sherif Monib : Data collection, data analysis

Adam Frampton : Editing

Simon Thomson : writing, editing

## Guarantor

Mr Nicholas Farkas

Mr Simon Thomson

## Declaration of competing interest

No conflicts of interest.
